# Clinical and Etiological Spectrum of Third, Fourth, Sixth, and Seventh Cranial Nerve Palsies: A Hospital-Based Cross-Sectional Study

**DOI:** 10.7759/cureus.82588

**Published:** 2025-04-19

**Authors:** Vidhya Verma, Priti Singh, Samendra Karkhur, Mahesh Verma

**Affiliations:** 1 Ophthalmology, All India Institute of Medical Sciences, Bhopal, Bhopal, IND; 2 Radiodiagnosis, Chirayu Medical College and Hospital, Bhopal, IND

**Keywords:** bell’s palsy, diabetes, diplopia, ocular cranial nerve palsy, trauma

## Abstract

Purpose: Impairment of the third, fourth, sixth, and seventh cranial nerves can lead to neuro-ophthalmic symptoms that severely affect visual function and quality of life. This study aimed to evaluate the clinical profile, etiological spectrum, and anatomical localization of ocular cranial nerve palsies involving the third, fourth, sixth, and seventh cranial nerves in patients presenting to a tertiary healthcare center in central India.

Materials and methods: This 12-month cross-sectional observational study involved 30 patients presenting with diplopia, headache, facial asymmetry, or restricted ocular movements. Patients aged 18 years or older diagnosed with palsy of the third, fourth, sixth, or seventh cranial nerve were included. A detailed history, neuro-ophthalmic examination, and imaging (CT or MRI) were performed. Data analysis was conducted using descriptive statistics, with categorical variables expressed as frequencies and percentages and continuous variables summarized as means and standard deviations.

Results: A total of 30 patients meeting the inclusion criteria were studied. Isolated seventh cranial nerve palsy was the most common, occurring in 12 patients (40%), which caused facial weakness and lagophthalmos. It was followed by isolated sixth cranial nerve palsy in 10 patients (33.3%), which led to diplopia and headache. Isolated third cranial nerve palsy was noted in five cases (16.7%). Mixed motor nerve palsies involving the third, fourth, sixth, and seventh cranial nerves were seen in three cases (10%). Notably, no patients presented with isolated trochlear (fourth nerve) palsy. Bell’s palsy, trauma, and diabetes were the leading causes.

Conclusions: Seventh cranial nerve palsy was the most common, with Bell’s palsy as the leading cause. Sixth cranial nerve palsy, primarily due to trauma-induced raised intracranial tension, was the second most frequent. Third cranial nerve palsy was mostly linked to diabetes, while cavernous sinus thrombosis was the main cause of multiple cranial nerve palsies. Larger studies are necessary to further validate these findings.

## Introduction

Ocular cranial nerve palsies involving the third, fourth, sixth, and seventh cranial nerves are significant causes of neuro-ophthalmic morbidity. These palsies present with diverse symptoms such as diplopia, strabismus, ptosis, and exposure keratitis, depending on the nerve affected. Dysfunction of the extraocular muscles disrupts ocular alignment and motility, which can impair binocular vision and contribute to visual disability. Additionally, facial nerve palsy compromises eyelid closure, which can lead to exposure keratopathy and corneal complications.

Globally, the prevalence of oculomotor nerve palsies varies. A population-based study from Olmsted County, USA, reported an incidence rate of 15 per 100,000 person-years for third and fourth cranial nerve palsies. Over a 15-year period, the age- and gender-adjusted annual incidence of sixth cranial nerve palsy was 11.3 per 100,000 individuals (95% CI: 9.3-13.2 per 100,000) [1]. Another hospital-based study found that microvascular ischemia, trauma, and neoplasms were the leading causes [2]. In India, data on ocular cranial nerve palsies are limited, with most information derived from neurological centers. A hospital-based study from India reported that sixth cranial nerve palsy (42.7%) was the most common, followed by third (34.7%) and fourth (17.7%) cranial nerve palsies. Ischemia caused most third and sixth cranial nerve palsies, while trauma was the leading cause of fourth cranial nerve palsy. Recovery rates were highest for third cranial nerve palsy (69.7%) and lowest for fourth cranial nerve palsy (45%) [3]. However, comprehensive ophthalmology-based data, particularly from central India, remain scarce.

This study aims to evaluate the clinical profile, etiologies, and demographic characteristics of patients presenting with third, fourth, sixth, and seventh cranial nerve palsies in a hospital-based population. It provides valuable insights into the region-specific disease burden and patterns, identifying associations between risk factors (such as diabetes, hypertension, and trauma) and specific cranial nerve involvement, as well as the frequency of isolated versus multiple cranial nerve involvement.

## Materials and methods

Study design and setting

This hospital-based, cross-sectional observational study was conducted over a one-year period (January 2023 to January 2024) at a tertiary care hospital in central India. The study adhered to the tenets of the Declaration of Helsinki (1964). It was approved by the Institutional Ethics Committee of All India Institute of Medical Sciences, Bhopal (approval number: AIIMS/BPL/RRB/Approval/2022/24, approval date: October 10, 2022). Informed consent was obtained from all participants before enrollment.

Study population

A total of 30 patients of both sexes, presenting with neuro-ophthalmic complaints either in the outpatient department or as inpatients, were included based on predefined inclusion and exclusion criteria. The inclusion criteria comprised patients aged 18 years or older with isolated or combined paralysis of the third, fourth, sixth, or seventh cranial nerves. Patients with congenital cranial nerve palsies, prior cranial nerve surgery or interventions, incomplete clinical records, or diagnoses of myasthenia gravis, myopathies, thyroid ophthalmopathy, or multiple sclerosis were excluded.

Sampling technique

A consecutive sampling technique was used to enroll all eligible patients presenting with neuro-ophthalmic symptoms such as diplopia, strabismus, lagophthalmos, ptosis, or restricted ocular movements. The diagnosis of cranial nerve palsies was confirmed through a detailed history and comprehensive clinical examination, including ophthalmologic and neurological assessments.

Data collection

Demographic data, including age, sex, and relevant systemic history such as hypertension, vascular diseases, diabetes mellitus, trauma, infections, and malignancies, were recorded for all participants.

Clinical examination

A comprehensive ophthalmologic examination was conducted, including best-corrected visual acuity, color vision assessment, evaluation of extraocular movements and diplopia charting, ptosis evaluation, ocular alignment assessment using cover-uncover and alternate cover tests, an anterior segment examination that included corneal sensation testing, and a fundus examination. Facial nerve function evaluation for seventh cranial nerve palsy was assessed using the House-Brackmann grading system to ensure standardized evaluation of facial nerve function. Neurological assessments included evaluation for mental status and cognitive function, cranial nerve function, motor and sensory systems, deep tendon reflexes, and plantar reflexes.

Clinical presentation and investigations

The type of nerve palsy, duration, and onset of symptoms, whether acute or chronic, were documented. Third nerve (oculomotor) palsy was assessed for ptosis, mydriasis, and ophthalmoplegia. Fourth nerve (trochlear) palsy was evaluated for vertical diplopia and head tilt, while sixth nerve (abducens) palsy was characterized by horizontal diplopia and esotropia. Additional investigations were performed as needed, including X-rays, B-scan ultrasonography, CT or MRI imaging, and cerebrospinal fluid analysis. Imaging of the skull, orbital fissures, and optic foramina was carried out when necessary to identify underlying structural causes. All patients underwent a comprehensive neuro-ophthalmic examination, which included assessments of pupillary responses, ocular motility, and cranial nerve function. Imaging studies, primarily contrast-enhanced MRI of the brain and orbits, were performed based on clinical indication. Lesions were evaluated for localization, enhancement patterns, and anatomical involvement relevant to cranial nerve pathways.

Statistical analysis

Data were compiled and analyzed using descriptive statistics. Continuous variables, such as age, duration, and clinical parameters, were expressed as means and standard deviations. Categorical variables, including etiology, nerve involvement, and risk factors, were presented as frequencies and percentages. For comparative analysis, the chi-square test was used to assess associations between etiology and nerve involvement, while logistic regression was applied to determine risk factors for specific nerve palsies. All statistical analyses were performed using SPSS Statistics version 25 (IBM Corp. Released 2017. IBM SPSS Statistics for Windows, Version 25.0. Armonk, NY: IBM Corp.).

## Results

Demographic profile

A total of 30 patients were examined, with a male preponderance. The male-to-female ratio was 1.3:1, and the mean age was 45.2 ± 12.7 years (range: 21-72 years). The age distribution of patients varied depending on the nerve involved. Patients with seventh cranial nerve palsy ranged between 22 and 70 years (mean age 41.6 years), with a male-to-female ratio of 2.5:1, while those with sixth cranial nerve palsy fell within the 28 to 67 years range. Third cranial nerve palsy was seen in a broader age group, spanning from 18 to 60 years old, whereas multiple cranial nerve palsies were identified exclusively in the 58 to 65 years range.

Seventh cranial nerve palsy primarily presented as a peripheral lower motor neuron (LMN) disorder. Sixth and third cranial nerve palsies were predominantly associated with lesions in the subarachnoid space. Cases involving multiple cranial nerve palsies were most commonly linked to pathology within the cavernous sinus.

Seventh cranial nerve palsy was the most common presentation, observed in 12 patients (40%), and was primarily characterized by unilateral facial weakness and lagophthalmos. Sixth cranial nerve palsy was noted in 10 patients (30%), who predominantly presented with diplopia and headache. Third cranial nerve palsy was associated with eyelid drooping and was observed in five patients (16.7%). The lowest incidence was recorded for multiple cranial nerve palsy, affecting three patients (10%). No cases of isolated fourth cranial nerve palsy were identified in this study.

Clinical and etiological profile

Seventh Cranial Nerve Palsy

Seventh cranial nerve palsy exhibited distinct etiological patterns, with trauma and inflammatory causes being more common in younger patients. In contrast, microvascular and ischemic etiologies were more frequently observed in older adults. The highest incidence was observed in the 40-49 age group. In the chi-square test results, χ² = 4.67, p = 0.031, a significant association was found between age and etiology. Bell’s palsy was identified as the most common etiological factor, accounting for 58.3% of cases (seven patients), followed by trauma in 25% of cases (three patients). Other causes included herpes zoster infection (8.3%) and ear infection (8.3%) (Table 1).

**Table 1:** Seventh cranial nerve palsy causes

Cause	Cases (n=12)	Percentage (%)
Bell’s palsy	7	58.3%
Trauma	3	25.0%
Ear infection	1	8.3%
Herpes zoster	1	8.3%

All cases were treated conservatively with corticosteroids, anti-inflammatory agents, and ocular surface lubrication. Exposure keratitis was seen in 42% of cases, requiring lubricants, nighttime patching, and regular corneal evaluations. Detailed statistical analysis of seventh cranial nerve palsy, showing significant associations between age and etiology, as well as key risk factors (Table 2).

**Table 2:** Logistic regression analysis for risk factors of seventh cranial nerve palsy * p < 0.05 indicates statistical significance OR: odds ratio, CI: confidence interval

Variable	OR	95% CI	p-value
Age ≥40 years	1.89	1.02–3.51	0.043*
Male sex	1.55	0.92–2.98	0.067
History of hypertension	2.12	1.04–4.37	0.038*
History of diabetes mellitus	2.45	1.08–5.21	0.027*
Trauma	1.76	0.85–3.27	0.059

Sixth Cranial Nerve Palsy

Sixth cranial nerve palsy accounted for 33.3% of cases, all of which presented with unilateral involvement. Isolated sixth cranial nerve palsy was the second most common isolated nerve palsy in this study, accounting for 10 cases (100%). The mean age of affected patients was 47 years, with an age range of 28 to 67 years. In the chi-square test results, χ² = 3.89, p = 0.048, a significant association was found between age and etiology. Trauma was identified as the leading etiological factor, observed in 40% of cases, followed by meningitis in 30%. Other causes included vascular pathology (20%) and idiopathic origin (10%) (Table 3). The study findings of sixth cranial nerve palsy show significant age-wise differences and risk factors (Table 4).

**Table 3:** Sixth cranial nerve palsy causes

Cause	No. of cases	Percentage
Trauma	4	40
Meningitis	3	30
Vascular	2	20
Idiopathic	1	10

**Table 4:** Logistic regression analysis for risk factors of sixth cranial nerve palsy * p < 0.05 indicates statistical significance OR: odds ratio, CI: confidence interval

Variable	OR	95% CI	p-value
Age ≥40 years	2.04	1.11–3.89	0.032*
Male sex	1.67	0.88–2.96	0.071
History of hypertension	1.89	1.02–3.54	0.041*
History of diabetes mellitus	2.22	1.14–4.32	0.029*
Trauma	1.94	1.02–3.21	0.044*

The most frequently reported symptom was diplopia, present in 40% of cases (four patients). A combination of headache and diplopia was noted in 20% (two patients), while another 20% (two patients) presented with only headache. The remaining 20% (two patients) did not report either symptom. Fundoscopic examination using a direct ophthalmoscope revealed papilledema in three out of 10 patients. Unilateral esotropia was the most common ocular finding, observed in 80% of cases (eight patients). The remaining 20% (two patients) had a parallel visual axis.

Third Cranial Nerve Palsy

Third cranial nerve palsy was seen in 16.7% of patients, all aged between 12 and 60 years, with a mean age of 34.8 years. Isolated third cranial nerve palsy was observed in all five patients (100%). The highest incidence was in the 10- to 19-year-old age group, with two cases. In the chi-square test results, χ² = 3.12, p = 0.077, which is not statistically significant, indicating no strong association between age and etiology. The male-to-female ratio was 4:1, and all cases involved unilateral palsy. The most common etiological factor was type 2 diabetes mellitus (two patients, 40%), followed by migraine, congenital causes, and vascular causes, each accounting for one case (Table 5). findings related to third cranial nerve palsy, highlighting key etiologies, age distributions, and associated risk factors (Table 6).

**Table 5:** Third cranial nerve palsy causes

Cause	No. of cases	Percentage
Diabetes mellitus	2	40
Migraine	1	20
Vascular	1	20
Congenital	1	20

**Table 6:** Logistic regression analysis for risk factors of third cranial nerve palsy * p < 0.05 indicates statistical significance OR: odds ratio, CI: confidence interval

Variable	OR	95% CI	p-value
Age ≥30 years	1.74	0.92–3.41	0.082
Male sex	2.25	1.11–4.39	0.041*
History of hypertension	1.98	1.06–3.82	0.037*
History of diabetes mellitus	3.12	1.52–6.42	0.014*

All five patients (100%) presented with ptosis. One patient (20%) reported a headache, while another (20%) experienced both a headache and diplopia. Three patients (60%) had neither headache nor diplopia.

Multiple Cranial Nerve Palsies

Multiple cranial nerve palsies were noted in 10% of patients, with two experiencing ophthalmoplegia. The mean age was 65 years (range: 58-65), including two males and one female. In the chi-square test results, χ² = 2.12, p = 0.14, no significant association was found between age and etiology. Drooping of the upper eyelid was noted in all cases. Headache occurred in two patients, while one had only diplopia. Causes were cavernous sinus thrombosis in 66.6% and diabetes mellitus in 33.3% (Table 7).

**Table 7:** Multiple cranial nerve palsy causes

Cause	No. of cases	Percentage
Cavernous sinus thrombosis	2	66.3
Diabetes mellitus	1	33.3
Trauma	0	0

Chi-square analysis did not show a statistically significant association between age and etiology (p = 0.112). Logistic regression identified diabetes mellitus and hypertension as significant risk factors (p < 0.05) (Table 8).

**Table 8:** Logistic regression analysis for risk factors of multiple cranial nerve palsy * p < 0.05 indicates statistical significance OR: odds ratio, CI: confidence interval

Variable	OR	95% CI	p-value
Age ≥50 years	1.86	0.92–3.72	0.094
Male sex	1.49	0.78–2.89	0.108
History of hypertension	2.11	1.06–4.18	0.043*
History of diabetes mellitus	2.98	1.42–5.89	0.017*

## Discussion

This hospital-based study highlights the clinical profile and causes of ocular cranial nerve palsies in patients presenting to a tertiary care ophthalmology department in central India. Seventh cranial nerve palsy with Bell’s palsy was the most frequently observed among all cranial nerve palsies evaluated in this study. Trauma and meningitis-related intracranial pressure elevation were the most common causes of sixth cranial nerve palsy. Third cranial nerve palsy was closely linked to diabetes mellitus, underscoring the need for better glycemic control in diabetic patients. Cavernous sinus thrombosis was the leading cause of multiple cranial nerve palsies, followed by diabetes mellitus.

Seventh cranial nerve palsy

Among all cranial nerve palsies studied, seventh cranial nerve palsy was the most frequently encountered, accounting for 40% of cases. All patients presented with unilateral involvement, consistent with existing literature [4].

The leading cause of seventh cranial nerve palsy in this study was Bell’s palsy, responsible for 58.3% of cases. This closely mirrors other study findings, where Bell’s palsy accounted for 55% of facial nerve palsy cases [4]. Our trauma incidence (25%) is slightly higher, which may be attributed to the increasing frequency of road traffic accidents in developing regions. All trauma-related facial palsies followed road traffic accidents, a trend also highlighted in trauma studies globally [5].

The age distribution of Bell’s palsy cases in our study, predominantly occurring between 20 and 50 years, is comparable to earlier findings by Zhang et al. (2020) [6]. However, our study found a male-to-female ratio of 2.5:1, which does not align with the near-equal gender distribution reported by Adour et al. [7]. This discrepancy may be due to the relatively small sample size in our study or regional variations in risk factors.

Three cases of facial nerve palsy were attributed to trauma, all resulting from road traffic accidents. This highlights the increasing contribution of traumatic nerve injuries in contemporary practice, particularly in developing regions with high rates of vehicular accidents. One case (8.3%) of facial nerve palsy secondary to a middle ear infection was noted, which corresponds well with the findings of Choi and Park (2015) [8], who also documented middle ear infections as a rare but significant cause of facial palsy. Similarly, herpes zoster infection was implicated in one case (8.3%), which is consistent with the 7% incidence reported in other studies [4].

The management of these patients primarily involved conservative treatment with anti-inflammatory medications, corticosteroids, and ocular surface protection. Notably, exposure keratitis was observed in 42% of patients with seventh cranial nerve palsy, necessitating the use of lubricating eye drops, ointments, and nighttime eye patching to prevent corneal complications.

Sixth cranial nerve palsy

Sixth cranial nerve palsy was the second most common ocular cranial nerve palsy, observed in 33.3% of cases. All patients had unilateral involvement. The predominant cause identified was trauma leading to raised intracranial pressure, which is distinct from earlier series like Rebelo et al. (2023), where 31.54% of cases were idiopathic [9]. Similarly, another study reported a substantial proportion of sixth cranial nerve palsies with unknown etiology, at 33% [2].

The vulnerability of the sixth cranial nerve to intracranial pressure changes is well established due to its long, tortuous course through the subarachnoid space. Increased intracranial pressure can compress the nerve between the pons and clivus or stretch it along the petrous ridge, leading to palsy. The trauma-related incidence of sixth cranial nerve palsy in our study (40%) was notably higher than that reported in other literature, such as Rebelo et al. (2023), who found trauma responsible for 19.5% of cases [9]. A more recent study by Patel et al. (2004) documented trauma in 12% of cases, indicating that regional factors, improved diagnostics, or changing patterns of trauma could account for the higher proportion observed in our cohort [1].

Third cranial nerve palsy

Third cranial nerve palsy was identified in 16.7% of cases. All patients were between 12 and 60 years, with a mean age of 34.8 years. This is broadly consistent with the 11 to 40 years peak incidence reported by other studies [1]. The gender distribution, however, skewed heavily toward males (male-to-female ratio of 4:1), differing from the equal gender ratio reported by some studies.

The most common cause of third cranial nerve palsy in our study was microvascular ischemia secondary to diabetes mellitus, a finding consistent with the results of Cahill et al. (2015) [10]. The right oculomotor nerve was affected in three cases, while the left was involved in two cases. This right-sided predominance has not been consistently reported in the literature and may be incidental given the small sample size. Diabetic third cranial nerve palsy arises from ischemic infarction within the core of the nerve trunk due to occlusion of nutrient arteries [11]. This mechanism is distinct from compressive or inflammatory causes, which more commonly affect the pupillary fibers, leading to pupillary involvement, a feature notably absent in diabetic cases.

Multiple cranial nerve palsies

Multiple cranial nerve palsies were diagnosed in 10% of patients, a slightly lower incidence than the 18.9% reported by Rebelo et al. (2023) [9]. The mean age of patients with multiple palsies was 65 years, and all had unilateral involvement. There was a slight male predominance, with a male-to-female ratio of 2:1.

The most common cause was cavernous sinus thrombosis (66.7%), with diabetes mellitus contributing to the remaining cases (33.3%). Cavernous sinus pathology can simultaneously affect cranial nerves III, IV, V1, V2, and VI due to their anatomical course through this venous sinus.

Anatomical localization

The anatomical localization of cranial nerve lesions in this study aligned with established anatomical vulnerabilities. All seventh cranial nerve palsies were of LMN origin, consistent with the nerve’s peripheral course [12]. Most sixth cranial nerve palsies were localized to the subarachnoid space, where the nerve’s lengthy intracranial path leaves it susceptible to intracranial pressure changes and stretch injury [13]. In cases of multiple cranial nerve palsies, the pathology was most often localized to the cavernous sinus, highlighting the importance of considering cavernous sinus pathology when multiple ocular motor nerves are simultaneously affected [14].

Strengths and limitations of the study

This prospective study on ocular cranial nerve palsies ensures robust and real-time data collection, which minimizes recall bias and strengthens the validity of the findings. By enrolling patients at the time of diagnosis and systematically documenting their clinical profiles, risk factors, and etiologies over a defined period, we establish clear temporal relationships between risk factors and outcomes. The study identifies the most common causes of ocular cranial nerve palsies in a hospital-based population, providing valuable epidemiological insights. It also establishes correlations between systemic conditions such as diabetes and hypertension with the occurrence of nerve palsies, reinforcing their role as potential risk factors. Additionally, the study contributes to improving early diagnosis and management strategies, equipping clinicians with evidence-based insights to refine diagnostic approaches and optimize patient care.

Despite its strengths, this study has certain limitations. Due to the small sample size, single-center design, and cross-sectional nature of the study, the findings may not be fully generalizable to broader populations. The limited follow-up duration restricts the assessment of long-term outcomes and potential recovery patterns. Additionally, there is a possibility of referral bias, as patients presenting to a tertiary care center may have more complex or severe presentations than those in primary or secondary healthcare settings. Future multi-center studies with extended follow-up can help validate our findings and further enhance the understanding of ocular cranial nerve palsies.

Larger, prospective, community-based, and hospital-based multi-center studies are recommended for broader applicability and to further validate these findings, providing a comprehensive understanding of the etiological patterns of ocular cranial nerve palsies. The findings of this study may aid in early diagnosis, guide targeted investigations, and inform management strategies for patients presenting with ocular cranial nerve palsies, thereby contributing to improved clinical outcomes and expanding current understanding in this domain.

## Conclusions

This study provides a comprehensive overview of the clinical profile, etiologies, and anatomical localization of ocular cranial nerve palsies in a hospital-based setting. The findings emphasize the importance of Bell’s palsy, trauma, and diabetes mellitus as leading causes across different cranial nerves. The high prevalence of diabetes and trauma-related palsies emphasizes the importance of managing systemic comorbidities and implementing injury prevention strategies.
